# Intentional Mindset Toward Robots—Open Questions and Methodological Challenges

**DOI:** 10.3389/frobt.2018.00139

**Published:** 2019-01-11

**Authors:** Elef Schellen, Agnieszka Wykowska

**Affiliations:** Social Cognition in Human-Robot Interaction, Istituto Italiano di Tecnologia, Genoa, Italy

**Keywords:** HRI (human robot interaction), intentional stance, humanoid robot, social cognition, social neuroscience

## Abstract

Natural and effective interaction with humanoid robots should involve social cognitive mechanisms of the human brain that normally facilitate social interaction between humans. Recent research has indicated that the presence and efficiency of these mechanisms in human-robot interaction (HRI) might be contingent on the adoption of a set of attitudes, mindsets, and beliefs concerning the robot's inner machinery. Current research is investigating the factors that influence these mindsets, and how they affect HRI. This review focuses on a specific mindset, namely the “intentional mindset” in which intentionality is attributed to another agent. More specifically, we focus on the concept of adopting the intentional stance toward robots, i.e., the tendency to predict and explain the robots' behavior with reference to mental states. We discuss the relationship between adoption of intentional stance and lower-level mechanisms of social cognition, and we provide a critical evaluation of research methods currently employed in this field, highlighting common pitfalls in the measurement of attitudes and mindsets.

## Introduction

Human-robot interaction (HRI) is an increasingly important topic, as robotic agents are becoming more developed, and are likely to take a conspicuous role as social agents in fields like health care and education, as well as daily living (Broadbent et al., 2009; Cabibihan et al., 2013). Humanoid robots, specifically, occupy a unique niche in our psychology (Wykowska et al., 2016; Wiese et al., 2017), as they are artificial human-like agents and thereby unlike any other natural social stimulus. Furthermore, they are distinctly different from other artificial agents such as virtual characters or avatars, in that they are embodied. Mapping out the way in which HRI differs from human-human interaction is essential for creating robots that successfully and efficiently interact with end-users. In this review, we will focus on a specific attitude or mindset that humans have toward other humans, namely the intentional stance (Dennett, 1987). We will consider the issue of adopting intentional stance toward artificial embodied agents (robots), we will discuss its relationship to other, lower-level mechanisms of social cognition, and we will critically evaluate methods to assess adoption of intentional stance. We posit that an intentional mindset, in which we frame a robot's actions in terms of its goals and desires, on the part of the user, might be crucial for well-functioning HRI.

## Intentional Stance in HRI

Dennett (1971, 1987) has proposed a categorization of different kinds of attitudes that humans have when explaining and predicting behavior of an observed system. This categorization includes three *stances* of differing complexity: the physical stance, the design stance and the intentional stance. These stances are used to heuristically make sense of events around us. In the *physical stance*, events are explained using physical rules of cause and effect; we know that water will boil when sufficiently heated, and that a ball rolling toward the edge of a table will fall down after crossing it. Using the *design stance*, one explains occurrences based on the assumption that these are outcomes of a purposefully designed system. This stance makes for an expedient framework facilitating interaction with systems that have input-output rules, like operating an industrial machine, where certain button presses correspond with certain machine operations. Finally, the *intentional stance* rests on the assumption that beliefs and desires underlie behavioral outcomes. Social robots occupy an ambiguous position in this framework, as they do not possess beliefs and desires in the folk psychology interpretation of these concepts. Still, they may be programmed to act as if they do, and adopting the intentional stance may result in the consequent interaction being more efficient and pleasant. The intentional stance, as it applies to HRI, shares many similarities with the concept of anthropomorphism. When a user ascribes human motivations and desires to a robot, in interpreting and predicting its behavior, they certainly anthropomorphize the robot. However, anthropomorphism seems to be a broader concept involving attribution of various human traits, while adopting the intentional stance refers more narrowly to adopting a strategy in predicting and explaining others' behavior with reference to mental states.

## Intentional Stance as a Crucial Factor in Activation of Mechanisms of Social Cognition in HRI?

Currently, research in the fields of social robotics and HRI is exploring the effect of attributing intentionality to robots, and the behavioral parameters of the robot that most efficiently induce this. In exploring the effect that attributing intentionality has on social interaction, participants of an experiment are sometimes led to believe that they are interacting either with a pre-programmed machine (e.g., Wykowska et al., 2014; Özdem et al., 2017) or with another human (who naturally has desires and beliefs). In some other studies, participants are first exposed to different agent types (e.g., human, humanoid robot, non-humanoid robot), and subsequently are led to believe that they are interacting with one of them (e.g., Krach et al., 2008). Inducing a particular (e.g., intentional) stance through instruction manipulation stands in contrast to methods used in research that aims to define the parameters under which participants spontaneously assume the intentional stance. Here, the intentional stance is induced through, for example, the robot's gaze, speech, or general behavior (e.g., Wykowska et al., 2015).

### Manipulating Adoption of Intentional Stance Through Explicit Instruction

Attitude or stance toward an artificial agent can be a major factor influencing the effectiveness of the subsequent interaction (e.g., Chaminade et al., 2012). Depending on beliefs concerning the processes underlying the observed behavior, people will use different strategies in interacting with them, and different brain regions will be activated during this interaction. An illustration of this comes from studies using economic games, in which participants were informed that they were playing a game together either with a computer or with another person (Kircher et al., 2009; Sripada et al., 2009). Here, participants use different strategies when playing the economic games; e.g., cooperating less readily in a prisoner's dilemma game and not engaging in punishing behavior in an ultimatum game when interacting with a machine (Rilling et al., 2004). On a neural level, brain regions associated with social cognition showed increased activation when participants believed they were engaging with a human as opposed to a machine (McCabe et al., 2001; Gallagher et al., 2002; Rilling et al., 2004). These results suggest that social cognitive mechanisms are more readily engaged in solving multi-agent problems, if the opponent is believed to be a human (see Schurz et al., 2014 for a meta-analysis). Although not all of these papers use Dennett's terminology, the belief that one's interaction partner is a human implies adoption of the intentional stance.

This trend extends beyond relatively high-level processes like decision making, since low-level psychological mechanisms such as sensory processing and attentional selection are similarly affected by whether one adopts the intentional stance or not (Wiese et al., 2012; Wykowska et al., 2014; Caruana et al., 2017). Studies have shown increases in neural signatures for sensory gain in response to social stimuli that are thought to be controlled by an intentional agent. Further, the effect of adopting the intentional stance on neuropsychological processes has been demonstrated in neuroimaging data. Brain regions related to mentalizing show more activation when participants interact with a robotic avatar that they believe is controlled by a person, than when they interact with this same avatar, but believe that its actions are preprogramed (Özdem et al., 2017). Activation of brain regions related to mentalizing have in turn been found to directly correlate with performance in tasks involving social cognition (Wiese et al., 2018).

These findings suggest that there are fundamental differences in how people interact with robots, based on the assumptions they hold concerning the cause of the robot's actions. Further, they demonstrate that interaction with non-human agents is positively influenced by the engagement of the brain's social capacities, which are increasingly activated when people believe that their interaction partner has a mind. Having adopted the intentional stance, humans will make use of the efficient information processing systems that social cognition offers, and social signals can be processed with increased efficiency. Social robots that are capable of inducing the intentional stance in their users might therefore be more capable of interacting with humans in a nigh-natural manner than those that cannot.

### Inducing Adoption of Intentional Stance Through Interaction

In research which explores the direct effects of adopting the intentional stance on social interaction, intentional stance acts as an independent variable and is mostly induced through belief manipulation, while robot behavior is identical in the experimental conditions. Parallel to this, ongoing research examines the ways that the intentional stance can be induced in a more spontaneous manner as a consequence of robot behavior (Terada et al., 2008; Yamaji et al., 2010). When the means of inducing the intentional stance is itself the topic of research, a means of quantifying the success of this process becomes necessary, and although the intentional stance as a concept is defined at length (Dennett, 1971, 1987), measuring its adoption still presents a challenge. Additionally, much of the literature on the induction of the intentional stance comes from the engineering disciplines, and has a different approach to the earlier discussed research, which is based in experimental psychology. Despite the apparent similarity in overall goals and terminology, the methodology and research questions in these respective fields are often quite discrepant. A particularly prominent type of paradigm in HRI research into intentional attitudes involves naturalistic and relatively open-ended experiments (e.g., Terada et al., 2007; Yang et al., 2015). Participants in these studies are generally not given strict instructions to perform a certain task with the robot in question, rather leaving the interaction to develop naturally. Despite the similarity between this type of setup and the envisaged goal environment of a robotic platform, experimental validity is often compromised this way, and questions about what aspect of the robot's behavior lead to the adoption of the intentional stance remain unanswered.

Finally, despite discussing the circumstances under which the intentional stance is adopted and the consequent role this stance plays in moderating social cognitive mechanisms separately, these phenomena, of course, do not exist in isolation. This is illustrated by Pfeiffer et al. (2011), who demonstrated that estimates of humanness concerning an on-screen avatar in a social gaze experiment varied with a combination of gaze behavior and subject expectations concerning the avatar. Estimates of humanness increased when the gaze behavior of the avatar seemed to fit with the strategy that it was pursuing, according to the task instruction. Therefore, it is important to note that ultimately, it is a combination of behavioral parameters of, and a person's expectations toward the robot that together inform estimates of humanness. Presently, this is rarely taken into consideration in HRI research.

## Methods to Assess Adoption of Intentional Stance

A major obstacle that affects all research into the parameters that induce adoption of the intentional stance is the lack of a reliable method to assess when people do in fact adopt the intentional stance. Specifically, to determine what behavioral parameters (e.g., Wykowska et al., 2015) induce intention attribution, it would be beneficial to develop a method that has the sensitivity to detect changes in attitudes resultant from robot behavior (i.e., in a pre-test vs. post-test manner). In this context, an interesting approach to measuring adoption of intentional stance has been reported by Thellman et al. (2017) or de Graaf and Malle (2018). In their paradigms, participants rated behaviors performed by either humans or robots in terms of, among other aspects, intentionality. The authors found that perceived intentionality of behaviors performed by robots, largely, closely matched that of identical behaviors performed by humans (Thellman et al., 2017; de Graaf and Malle, 2018). Note, however, that these findings come from surveys in which participants consider behaviors based on either written scenarios or pictures, and therefore reflect a conceptual consideration of the behavior, and not a direct reaction to it (Fussell et al., 2008).

Other methods have been used with varying levels of subtlety, few of which have undergone thorough validation. This lack of a common criterion makes comparison of studies on this topic especially troublesome, and precludes the use of common quantitative meta- analyses such as the computing of effect sizes (see Steinfeld et al., 2006; Weiss and Bartneck, 2015 for exceptions). Some researchers infer adoption of intentional stance indirectly by means of a different variable, which might be vulnerable to the influence of confounding variables, for instance, by the degree of successful interaction with a robot (Yamaji et al., 2010). Here, the authors found that children would often deposit trash into mobile trash cans when these trash cans moved toward litter on the ground and make twisting and bowing movements, signaling that this litter needed to be disposed of. Yamaji et al. (2010) concluded that this constituted a successful induction of intention attribution, as users were able to interpret what action was required from them. Alternatively, intention attribution has been inferred by the degree to which participants reported feeling “deceived” by a robot displaying unexpected behavior during an interactive game, which increased the likelihood that the robot would win (Terada and Ito, 2010). These authors argued that feeling deceived implies attributing intention to the robot.

The most intuitive method to measure the adoption of intentional stance involves simply asking participants to reflect on their interaction with a robot (Terada et al., 2008). Sirkin et al. (2015) deduced the degree of intentionality that participants attributed to the robot from a semi-structured interview. Terada et al. (2007) took a more straightforward approach, simply asking participants which of Dennett's stances they adopted in a brief interaction with a remote controlled chair. This method has its drawbacks, in that it requires introspection on the part of the participants as well as a moderate level of knowledge concerning Dennett's terminology. Introspection, though, is a notoriously unreliable research tool (Nisbett and Wilson, 1977; Fiala et al., 2014). Other studies make use of unstructured interviews, and subject the respondents' answers to analysis (Sirkin et al., 2015). Despite not requiring the deep level of introspection that is involved in asking participants to describe which of Dennett's stances they have adopted, this measure is still both explicit and reliant on self-reporting. Furthermore, being a qualitative technique, it does not lend itself well to comparative analysis between conditions or between studies.

**Figure 1:**
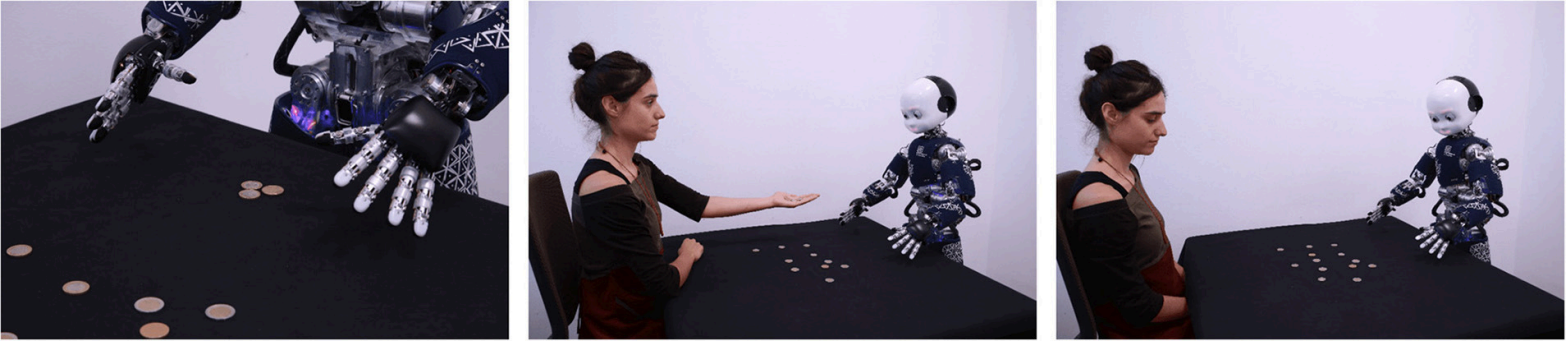
Example scenario from the questionnaire of Marchesi et al. (2018).

Questionnaires are a common tool to obtain a quantitative measure of attitudes toward a robot, which avoids the pitfalls of the earlier described techniques. Questionnaires in HRI typically cover multiple aspects concerning the robot's perceived likability, utility, and humanness/anthropomorphism. Examples are the Psychological Humanoid Impression iTems (PHIT, Kamide et al., 2013), the Godspeed Questionnaire Series (GSQ, Bartneck et al., 2009) and the Robotic Social Attributes Scale (RoSAS, Carpinella et al., 2017). None of these are specifically aimed at assessing the adoption of the intentional stance toward a robot, though the PHIT has a subscale (Agency) that relates to the robot having agency and intentions of its own, although this subscale has not been explicitly validated as a corollary of adopting the intentional stance (Koda et al., 2016). A novel questionnaire has been designed specifically to assess the adoption of the intentional stance in HRI (Marchesi et al., 2018). This questionnaire consists of short series of photographs depicting the iCub robot (Metta et al., 2010) in various action sequences, see Figure 1 for example scenario. The questionnaire provides two descriptions of the events in each series, one offering an explanation in terms of the mechanics and design of the robot, and the other in terms of intentions, beliefs and desires. Participants are asked to choose between the two alternative interpretations of the depicted scenario.

The story line presented in the scenarios usually does not offer a unique interpretation, and has an “open ending.” In the example given, the description below the photographs reads:

“iCub receives three coins. iCub looks at the three coins.

1. Because iCub thinks it deserves more
2. Because iCub is counting.”

The utterance was designed to be ambiguous in order to make each interpretation (mentalistic: option 1 in the above example, vs. mechanistic: option 2 in the above example) equally plausible. This questionnaire offers a dedicated research tool that does not necessitate introspection or knowledge about Dennett's taxonomy of stances on the part of the respondents, and is to some degree implicit, in that it does not directly ask participants to pass judgment on the robot's qualities. Still, this method has its drawbacks. The questionnaire's items depict one type of robot. This limits its generalizability, given the large degree of heterogeneity with which different robotic platforms are perceived (Mathur and Reichling, 2016). Additionally, it relies on conceptual judgments of a depicted behavior and does not reflect mentalizing processes that arise from a direct interaction with a robot.

Ideally, future research will be able to offer a still more implicit measure of intentional stance adoption. This may come in the form of paradigms that analyze reaction time and accuracy to assess underlying attitudes or stances, much like the implicit association test (Greenwald and Banaji, 1995; Luo et al., 2006). Additionally, research into neuropsychological markers and correlates of the intentional stance can explore the neural basis of assuming the intentional stance, offering ways to circumvent behavioral measures altogether (Spunt et al., 2015). Having a reliable technique to measure adoption of the intentional stance which can be compared across experiments, combined with a more methodical and controlled approach in studies attempting to parametrize robot behavior that gives rise to adoption of intentional stance, will help toward creating a comprehensive taxonomy of effective robot social behavior.

## Conclusion

In sum, adoption of intentional attitudes in HRI is a topic to be addressed, as it might facilitate and enhance the effectiveness of social cognitive mechanisms, and thereby the overall quality and efficiency of human-robot communication. This has implications for robot design, as robots that are able to spontaneously induce intentional attitudes in their users will most likely be more effective communicators. Current research into robot behavioral parameters that induces this intentional attitude in users has certain limitations. For example, the lack of a reliable and common method to measure the adoption of intentional attitudes seriously impedes the validity of experimental findings and the comparability of different studies. Without a consensus on what constitutes adoption of the intentional stance and how one can quantify this, researchers in the field of HRI will be using different measuring rods. We propose that after having established well-validated methods for measuring adoption of the intentional stance, it would be of great importance to understand the conditions under which the intentional stance is adopted toward artificial agents.

## Author Contributions

ES performed literature research and review. AW conceived of the article's topic and structure, revised the manuscript and provided feedback with guidance on its writing.

### Conflict of Interest Statement

The authors declare that the research was conducted in the absence of any commercial or financial relationships that could be construed as a potential conflict of interest.
